# Transcriptome Sequencing of Diverse Peanut (*Arachis*) Wild Species and the Cultivated Species Reveals a Wealth of Untapped Genetic Variability

**DOI:** 10.1534/g3.115.026898

**Published:** 2016-10-10

**Authors:** Ratan Chopra, Gloria Burow, Charles E. Simpson, Jennifer Chagoya, Joann Mudge, Mark D. Burow

**Affiliations:** * Department of Plant and Soil Sciences, Texas Tech University, Lubbock, Texas 79409; † United States Department of Agriculture, Agriculture Research Service, Cropping Systems Research Laboratory, Lubbock, Texas 79415; ‡ Texas A&M AgriLife Research, Stephenville, Texas 76401; § Texas A&M AgriLife Research, Lubbock, Texas 79403; ** National Center for Genome Resources, Santa Fe, New Mexico 87505

**Keywords:** groundnut, alleles, SNPs, domestication, evolution

## Abstract

To test the hypothesis that the cultivated peanut species possesses almost no molecular variability, we sequenced a diverse panel of 22 *Arachis* accessions representing *Arachis hypogaea* botanical classes, A-, B-, and K- genome diploids, a synthetic amphidiploid, and a tetraploid wild species. RNASeq was performed on pools of three tissues, and *de novo* assembly was performed. Realignment of individual accession reads to transcripts of the cultivar OLin identified 306,820 biallelic SNPs. Among 10 naturally occurring tetraploid accessions, 40,382 unique homozygous SNPs were identified in 14,719 contigs. In eight diploid accessions, 291,115 unique SNPs were identified in 26,320 contigs. The average SNP rate among the 10 cultivated tetraploids was 0.5, and among eight diploids was 9.2 per 1000 bp. Diversity analysis indicated grouping of diploids according to genome classification, and cultivated tetraploids by subspecies. Cluster analysis of variants indicated that sequences of B genome species were the most similar to the tetraploids, and the next closest diploid accession belonged to the A genome species. A subset of 66 SNPs selected from the dataset was validated; of 782 SNP calls, 636 (81.32%) were confirmed using an allele-specific discrimination assay. We conclude that substantial genetic variability exists among wild species. Additionally, significant but lesser variability at the molecular level occurs among accessions of the cultivated species. This survey is the first to report significant SNP level diversity among transcripts, and may explain some of the phenotypic differences observed in germplasm surveys. Understanding SNP variants in the *Arachis* accessions will benefit in developing markers for selection.

Domesticated peanut (*Arachis hypogaea* L.) is one of many polyploid species belonging to the genus *Arachis*, family Fabaceae, and is native to South America; many of these species come from a region including Brazil, Bolivia, and Paraguay (Gregory *et al.* 1980). There are 80 species, including diploids and tetraploids, described in the genus, categorized into nine sections according to morphology and crossability (Krapovickas and Gregory 1994). *A. hypogaea L*. is classified into two subspecies, *hypogaea* and *fastigiata*, based on the presence or absence of flowers on the main axis, and spreading or erect growth habit. These two subspecies are further classified into six botanical varieties based on morphology (Krapovickas and Gregory 1994; Valls and Simpson 2005; Lavia *et al.* 2008).

The origin of *A. hypogaea* L. and identity of progenitor species have been of interest to plant taxonomists, geneticists, and breeders. However, our knowledge of the origin of cultivated peanut is limited compared with other major crops. More than eight diploid species having either the A- or B- genome have been considered to be involved in the origin of peanut (Norden 1973; Gregory and Gregory 1976; Kochert *et al.* 1991, 1996; Fernandez and Krapovickas 1994; Krapovickas and Gregory 1994; Lavia 1998; Raina and Mukai 1999; Raina *et al.* 2001; Moretzsohn *et al.* 2004; Seijo *et al.* 2007; Bertioli *et al.* 2011). More recently, Seijo *et al.* (2007) and Bertioli *et al.* (2011, 2016) provided stronger evidence of *A. duranensis* and *A. ipaënsis* being the progenitor species of modern cultivars.

All molecular studies, even using older types of molecular markers, of wild peanut species have identified significant molecular-level variability among these accessions (Halward *et al.* 1991; Lu and Pickersgill 1993; Kochert *et al.* 1991, 1996). Wild *Arachis* species possess genetic variability in pest and disease resistance traits, which could be used to improve the cultivated peanut (Stalker and Moss 1987). Alleles that confer resistance to pests and disease in some wild *Arachis* species have been successfully transferred into cultivated peanut (Simpson 2001; Mallikarjuna *et al.* 2011).

In contrast, many molecular studies have demonstrated no or little genetic variability in the cultivated species, *A. hypogaea*. This was first noticed with the use of allozyme and RFLP markers (Halward *et al.* 1991; Kochert *et al.* 1991, 1996; Lu and Pickersgill 1993; Burow *et al.* 2009), which demonstrated an almost complete lack of genetic diversity among the cultivated peanut accessions. It was concluded that a genetic bottleneck occurring as a result of the polyploidization event, coupled with a self-pollinating reproductive system, and the use of a few elite breeding lines with little exotic germplasm in breeding programs, has resulted in a narrow genetic base of peanut cultivars. Natural gene exchange between wild diploid species and cultivated peanut may have been limited due to genomic rearrangement as well as differences in ploidy levels (Soltis and Soltis 1999; Huang *et al.* 2012). Since then, >10,000 SSR markers have been identified in peanut, many solely among wild species, but few SSR marker maps possess 200 or more SSR markers, again suggesting low genetic variability in the cultivated species.

Despite the results of some molecular studies, phenotypic evaluation of germplasm collections, such as core collections of 1704 (ICRISAT), 831 (United States), and 582 (China) accessions (Upadhyaya 2003; Holbrook *et al.* 1993; Jiang *et al.* 2004), and minicore collections (Upadhyaya *et al.* 2002; Holbrook and Dong 2005) point to a different conclusion. Evaluation has demonstrated significant phenotypic diversity for numerous traits, including resistance to leaf spots, tomato spotted wilt virus, other biotic stresses, for tolerance to drought or heat stress, and for early maturity (Isleib *et al.* 1995; Anderson *et al.* 1996; Upadhyaya 2003, 2005, Upadhyaya *et al.* 2006a,b; Selvaraj *et al.* 2011; Wang *et al.* 2011a; Jiang *et al.* 2014; Pandey *et al.* 2014; Singh *et al.* 2014). To date, these have not been accompanied by molecular characterization at SNP levels.

Technology for DNA sequencing and SNP analysis has made great progress recently, both for high throughput and for low cost per sequence. Due to the ubiquity of SNPs, and the far greater power to identify polymorphisms than other types of marker analysis, sequencing is able to identify genetic diversity better than other marker types. RNASeq allows transcriptome profiling and SNP identification with high read depth at much lower cost than whole genome sequencing. Annotation of assembled RNASeq reads can help in understanding the function of a gene or a transcript. Annotations have been used in several crops for establishing genetic information where limited knowledge exists (Garg *et al.* 2011; Li *et al.* 2012). SNPs in annotated transcripts associated with traits would benefit functional characterization of genes. For example, a mutation in the fatty acid desaturase FAD2B gene in peanut is being used for selection of the high-oleic trait (Wang *et al.* 2011b). Use of RNASeq on elite peanut genotypes has provided evidence of large numbers of SNPs among accessions (Chen *et al.* 2013; Zhang *et al.* 2012; Chopra *et al.* 2015); such a large number of polymorphisms were not known using earlier molecular marker techniques (Kochert *et al.* 1996).

Validation of SNPs identified by sequencing is required for use of these SNPs in generation of molecular maps or association with specific traits. Validation of SNPs in crops such as soybean (Wu *et al.* 2011), peanut (Nagy *et al.* 2012; Khera *et al.* 2013), or chickpea (Deokar *et al.* 2014) has benefited in producing high-density maps extended to QTL and GWAS analysis. Assembly, annotation, and validation of the information generated using RNASeq is an integrated process needed for application of datasets. Integrating these processes is becoming routine, as it is advantageous for a program to develop its own resources to address research goals of a particular crop.

The hypothesis of the current work is that, commensurate with the phenotypic differences observed in the field, there is actually a substantial amount of unrealized genetic diversity in peanut at the nucleotide level (both among the diploid and tetraploid species accessions). Although recent studies at the genome levels were carried out in two progenitor wild species (Bertioli *et al.* 2016), there is no survey of SNP level variability among the broader array of wild species, nor among cultivated accessions. Therefore, in this present study, a collection of eight cultivated tetraploid accessions, eight wild diploids, a natural amphidiploid, and a synthetic amphidiploid were selected for transcriptome analysis, adding in also data from four previously sequenced cultivated accessions (Chopra *et al.* 2015). The objectives of the study were (a) to measure allelic and transcript diversity among the accessions of different species within the genus *Arachis*, (b) to estimate number of genes in peanut, (c) to compare to the expected phylogenic relationships of the *Arachis* accessions, (d) to highlight SNPs specific to one accession or to a group of market types or to a group of different genome affiliations, and (e) to validate the SNPs obtained from the bioinformatics calls that can be used in a breeding program.

## Materials and Methods

### Plant accessions

A total of 12 tetraploid accessions from *A. hypogaea* was used, 10 tetraploids representing the three botanical types cultivated in the United States, namely subsp. *hypogaea* var *hypogaea*, subsp. *fastigiata* var *fastigiata*, and subsp. *fastigiata* var *vulgaris*, and two tetraploids representing other botanical types namely, subsp. *hypogaea* var *hirsuta*, and subsp. *fastigiata* var *aequatoriana* (Table 1). *A. monticola* is the only known wild tetraploid relative in section *Arachis*, and was included for comparison to the cultivated peanut and potentially for understanding gene flow between *A. monticola* and *A. hypogaea*. Eight wild diploid accessions, including probable parental species *A. duranensis* and *A. ipaënsis* of *A. hypogaea* were also included. Use of these latter accessions provided a platform for assessing the extent of genetic variability among the wild species. A synthetic amphidiploid, TxAG-6 (Simpson *et al.* 1993) was also included in the study (Table 1); parents of this were *A. cardenasii*, *A. diogoi*, and *A. batizocoi*. TxAG-6 has donated alleles that have resulted in release of four nematode-resistant cultivars, namely COAN (Simpson and Starr 2001), NemaTAM (Simpson *et al.* 2003a,b), Tifguard (Holbrook *et al.* 2008), and Webb (Simpson *et al.* 2013).

**Table 1:** Description of *Arachis* accessions utilized for the sequence analysis: their origin and ploidy levels

Accession	Species	Genome	Origin	Botanical Type	References
GKP10017	*Arachis cardenasii*	AA	Bolivia	Wild	Krapovickas *et al.* (1994)
GK10602	*Arachis diogoi*	AA	Bolivia	Wild	Krapovickas *et al.* (1994)
K7988	*Arachis duranensis*	AA	Argentina	Wild	Krapovickas *et al.* (1994)
KSSc38901	*Arachis duranensis*	AA	Bolivia	Wild	Krapovickas *et al.* (1994)
GKPSSc30076	*Arachis ipaënsis*	BB	Bolivia	Wild	Krapovickas *et al.* (1994)
GKSSc30097	*Arachis magna*	BB	Bolivia	Wild	Krapovickas *et al.* (1994)
K9484	*Arachis batizocoi*	KK	Bolivia	Wild	Krapovickas *et al.* (1994)
KSSc36024	*Arachis cruziana*	KK	Bolivia	Wild	Krapovickas *et al.* (1994)
GKBSPSc30062	*Arachis monticola*	AABB	Argentina	Wild	Krapovickas *et al.* (1994)
BSS56	*Arachis hypogaea*	AABB	West Africa	Spanish	Burow *et al.* (2014)
Florunner (UF439-16-10-3-2)	*Arachis hypogaea*	AABB	Florida	Runner	Norden *et al.* (1969)
Jupiter	*Arachis hypogaea*	AABB	Oklahoma	Virginia	—
New Mexico Valencia C	*Arachis hypogaea*	AABB	New Mexico	Valencia	Hsi (1980)
OLin	*Arachis hypogaea*	AABB	Texas	Spanish	Simpson *et al.* (2003)
PI502111 (COC155B)	*Arachis hypogaea*	AABB	—	Virginia	Holbrook and Dong (2005)
PI290538 (COC224)	*Arachis hypogaea*	AABB	India	Runner	Holbrook and Dong (2005)
PI268868 (COC367)	*Arachis hypogaea*	AABB	Sudan	Virginia	Holbrook and Dong (2005)
PI158854 (COC559)	*Arachis hypogaea*	AABB	China	Valencia	Holbrook and Dong (2005)
PI648241	*Arachis hypogaea*	AABB	Ecuador	Hirsuta	—
PI648242	*Arachis hypogaea*	AABB	Peru	Aequatoriana	—
Tamrun OL07	*Arachis hypogaea*	AABB	Texas	Runner	Baring *et al.* (2006)
TxAG-6	—	AAKK	Texas	Synthetic tetraploid	Simpson (1993)

### Sequences, de novo assembly and annotation

Plants were grown in the greenhouse at Texas A&M AgriLife Research. Unopened leaves, roots, and pods from yellow, brown, and black pod maturity stages were selected for RNA isolation. RNA was isolated using the Trizol reagent (Invitrogen, Grand Island, NY). Accessions OLin, Tamrun OL07, Jupiter, and New Mexico Valencia C were sequenced on an Illumina GAIIx using 2 × 54 paired-end reads as reported previously (Chopra *et al.* 2015). The remaining 18 genotypes were sequenced on an Illumina HiSeq 2000 instrument using 2 × 50 paired end reads at the National Center for Genome Resources, Santa Fe, NM. *De novo* assemblies were performed for all 22 genotypes using the Trinity (Grabherr *et al.* 2011) assembler, with the parameters used in Chopra *et al.* (2014) for the peanut transcriptome. Assembly statistics from Trinity, which enumerate unique transcripts as an estimate of the number of genes in a transcriptome, and the measure of the total number of structural variants derived from genes, are reported.

A consensus assembly was built by pooling transcripts of all 22 accessions, followed by elimination of duplicate transcripts using CAP3 (Huang and Madan 1999) at a minimum match of 95%. Annotations of the consensus assembly were performed against the *nr* database from NCBI, and from the Swissprot and Trembl databases from UniProt (UniProt Consortium 2015). Sequences from the consensus assembly were assigned gene ontology (GO) categories using the Mercator web-based tool (Lohse *et al.* 2014). Brief annotations of the transcripts in the consensus assembly assigned to different biological and molecular functions by Mercator are provided in Supplemental Material, File S1. Transcripts assigned to different transcription factor families are provided in File S2.

### Mapping and variant calling

Raw reads of all samples were aligned back to the OLin transcriptome sequence (Chopra *et al.* 2014) using the bwa.0.7.5a (Li and Durbin 2010) aligner, using the backtrack approach. These aligned reads were further processed using default values of GATK tools (DePristo *et al.* 2011) to improve the qualities of the alignment process. Aligned files were sorted and indexed using Picard tools (http://broadinstitute.github.io/picard/). The GATK variant calling tool was used to identify SNPs and indels among these genotypes using default parameters for UnifiedGenotyper at a ploidy level of 2. Parameters of MQ (Mapping Quality) > 40.0, AF (Allele Frequency) > 0.10, and DP (Read Depth) > 50 were used to filter the SNP calls using vcftools (Danecek *et al.* 2011). Variant statistics were calculated using the vcf-stats script, which reports variants among the samples used for comparisons.

### Diversity and population structure

Using SNPs identified from sequences that had a read depth of at least 50, and variant allele frequency >10% across 22 genotypes, principal components analysis (PCA) was performed using the *R* package SNPRelate (Zheng *et al.* 2012). The SNP datasets obtained after using parameters to filter the SNPs were used to calculate the individual dissimilarities for each pair of genotypes using the command snpgdsHCluster[snpgdsDiss(genofile, num.thread = 2, autosome.only = FALSE)]. A dendrogram was generated for each of the paired accessions after 5000 permutations using the command snpgdsCutTree(“FileName,” n.prem = 5000).

### SNP validation

Genomic DNA was prepared from seed tissue of the 12 sequenced accessions (File S3), including treatment with RNaseA during homogenization, and purified using the Qiagen DNAeasy miniprep kit (Qiagen, Valencia, CA). Sixty-six pairs of primers (File S4) were designed to perform validation of allele calls in the sequenced genotypes. For each putative SNP, two allele-specific forward primers, and one common reverse primer were designed (LGC Genomics, Hoddesdon, UK).

Genotyping reactions were performed on a LightCycler 480 (Roche, Branford, CT) in a final volume of 10 µl containing 1× KASP Reaction Mix (LGC Genomics, Hoddesdon, UK), 0.14 µl assay mix, and 10–20 ng of genomic DNA. The following cycling conditions were used: 15 min at 94°; 10 touchdown cycles of 20 sec at 94°, 60 sec at 65–57° (dropping 0.8° per cycle); and 26 cycles of 20 sec at 94°, 60 sec at 57°, and read at 37° for 5 sec. Fluorescence detection of the reaction was performed using a built-in scanner and the data were analyzed using the LightCycler 480 software (Roche, Branford, CT).

### Phasing approach

To resolve homeologs in the peanut tetraploid reference sequence, we used the phasing approach. Genome-specific assemblies in tetraploid peanut using the phasing approach were generated by aligning OLin raw reads to the OLin reference transcriptome (60,798 contigs) using the BWA backtrack approach. Polymorphisms among the mapped reads were detected using the GATK software as it has been shown to perform well on RNAseq data (McCormick *et al.* 2015). Called SNPs and Multiple Nucleotide Polymorphisms (MNPs) were phased using the HapCUTv.0.5software (Bansal and Bafna 2008) with default parameters. To generate homeolog-specific subassemblies, we used the strategy employed by Krasileva *et al.* (2013), sorting the reads within each phased SNP block based on the HapCUT output, and reassembling *de novo* the reads for each block and phase using parallelized runs. Haplotype information along with bam files were passed through readphaser to sort the reads within each block into phases based on HapCUT tables, and reassembled.

To evaluate the resolution of the assemblies generated from each of these approaches, we mapped the raw reads back to each assembly. SNPs were called on each of these assemblies using GATK. Parameters used to evaluate assemblies were the number of heterozygous SNPs, homozygous SNPs, and number of SNPs per transcript.

### Data availability

BAM files are deposited in SRA database under bioproject PRJNA248910: SRR1534396, SRR1535034, SRR1535035, SRR4039437, SRR4039359, SRR4039358, SRR4039357, SRR4039017, SRR4039016, SRR4039015, SRR4038966, SRR4038965, SRR4038285, SRR4037988, SRR4038281, SRR4038254, SRR4038252, SRR4038060, SRR4037987, SRR4037985, SRR4037986, and SRR4037959. *De novo* assemblies and annotations described in this manuscript have been deposited at Figshare at the following URLs: https://dx.doi.org/10.6084/m9.figshare.3580650.v2, https://dx.doi.org/10.6084/m9.figshare.3580656.v2, https://dx.doi.org/10.6084/m9.figshare.3580659.v1, and https://dx.doi.org/10.6084/m9.figshare.3580662.v1.

## Results

### Over 500 million reads provided a large dataset for de novo assembly and annotation

A total of 539 million filtered reads were obtained from 22 accessions including wild species plus cultivars and landraces of *A. hypogaea*, including different subspecies, and originating from different continents (Table 2). For diploids, the number of raw reads ranged from 13.8 to 24.5 million, and for tetraploids, the number of reads ranged from 18.4 to 43.4 million. *De novo* assemblies of filtered reads of diploid accessions using Trinity generated an average of 38,903 transcripts, and 27,045 unique transcripts (Table 2) of size >200 bp. Tetraploid *de novo* assemblies generated an average of 51,744 total transcripts and 29,234 unique transcripts. Overall the number of genes per diploid peanut genome averaged 28,321 based on the assemblies of 22 accessions.

**Table 2:** *De novo* assembly characteristics of sequences from twenty-two transcriptomes

Genotype	Reads	Total Transcripts*^a^*	Unique Transcripts*^b^*	N50
GKP10017	16,015,713	37,767	27,745	1333
GK10602	24,501,057	44,635	27,406	1484
K7988	18,698,563	39,088	27,219	1393
KSSc38901	16,206,929	37,379	25,530	1401
GKPSSc30076	16,774,125	31,800	26,102	1107
GKSSc30097	18,264,048	34,673	26,808	1238
K9484	20,009,991	41,750	28,287	1405
KSSc36024	13,852,235	37,654	27,261	1159
GKBSPSc30062	16,366,546	37,944	26,654	1184
BSS56	23,249,778	39,343	26,735	1294
Florunner (UF439-16-10-3-2)	18,498,900	39,349	27,226	1253
Jupiter	43,494,034	74,615	31,184	1687
New Mexico Valencia C	43,327,345	71,264	30,677	1655
OLin	38,335,246	67,098	30,673	1641
PI502111(COC155B)	22,844,455	41,513	26,932	1370
PI290538 (COC224)	19,126,674	39,916	27,174	1200
PI268868 (COC367)	25,275,018	38,983	25,079	1401
PI158854 (COC559)	31,318,101	47,007	27,038	1419
PI648241	27,952,734	43,763	27,327	1355
PI648242	23,770,118	44,206	27,146	1405
Tamrun OL07	41,601,127	79,214	44,760	1535
TxAG-6	19,865,766	46,399	28,089	1359

a Number of total transcripts including splice variants, homeolog and paralog copies assembled using the *de novo* assembly approach for each of the accession.

b Number of unique transcripts that were in each genotype excluding the derivatives such as splice variants/homeologs.

On merging the transcripts in diploids and tetraploids, we observed there was an increase in the total number of transcripts in each pool (Table 3). The resultant A-, B- and K- genome pooled assemblies had from 35,530 to 57,851 transcripts each. The A- genome pool had about 45% or more transcripts than the *de novo* assembly of a single A-genome species. The B- and K- genome pools had slightly lower ratios of pooled transcripts compared to individual species accessions (Table 4). For the tetraploid genotypes of *A. hypogaea*, assembled transcripts were merged, and 100,328 transcripts were obtained.

**Table 3:** Consensus assemblies generated from CAP3 for genome-specific pools

Assembly	No. of Contigs	N50 (bp)	Average Contig Length
A_Genome_Diploids*^a^*	57,851	1700	1145
B_Genome_Diploids*^a^*	35,530	1362	910
K_Genome_Diploids*^a^*	43,462	1518	993
*Arachis_hypogaea*	100,328	1816	1205
*Arachis_monticola*	37,944	1184	798
TxAG-6	46,399	1359	903
Arachis_Pooled_Consensus	165,892	1874	1277

a Assemblies pooled for each genome affiliates for estimating transcript diversity in each pool or in the *Arachis* genus.

**Table 4:** Variants in each accession were identified in comparison to OLin as the reference

	Genotype	No. of Transcripts with SNPs	No. of Homozygous Variants*^a^*	Average No. of SNPs per Transcript*^b^*	Number of SNPs per kb*^c^*
Diploids	GKP10017	22,192	117,812	5.31	1.57
	GK10602	22,564	106,882	4.74	1.43
	K7988	21,931	122,370	5.58	1.64
	KSSc38901	21,693	117,252	5.41	1.57
	GKPSSc30076	18,475	96,241	5.21	1.28
	GKSSc30097	21,638	109,797	5.07	1.47
	K9484	24,929	168,289	6.75	2.25
	KSSc36024	24,064	157,444	6.54	2.11
Tetraploids	GKBSPSc30062	6963	14,340	2.06	0.19
	BSS56	5326	9012	1.69	0.12
	Florunner (UF439-16-10-3-2)	5557	9454	1.7	0.12
	Jupiter	1844	3318	1.8	0.04
	New Mexico Valencia C	2146	3451	1.61	0.04
	PI502111(COC155B)	5361	9468	1.77	0.12
	PI290538 (COC224)	6068	10,832	1.79	0.14
	PI268868 (COC367)	4715	8491	1.8	0.11
	PI158854 (COC559)	4306	7209	1.67	0.09
	PI648241	4669	8212	1.76	0.11
	PI648242	4084	7081	1.73	0.09
	Tamrun OL07	2263	3923	1.73	0.05
	TxAG-6	22,607	87,574	3.87	1.17

Note: OLin is not reported here as the above comparisons were made relative to OLin transcriptome.

a Number of homozygous SNPs present in each accession relative to OLin transcriptome.

b Average number of transcripts calculated using Number of homozygous SNPs/Number of transcripts in an accession.

c SNP rate calculated based on pair-wise comparison of each accession with OLin.

**Figure 1:**
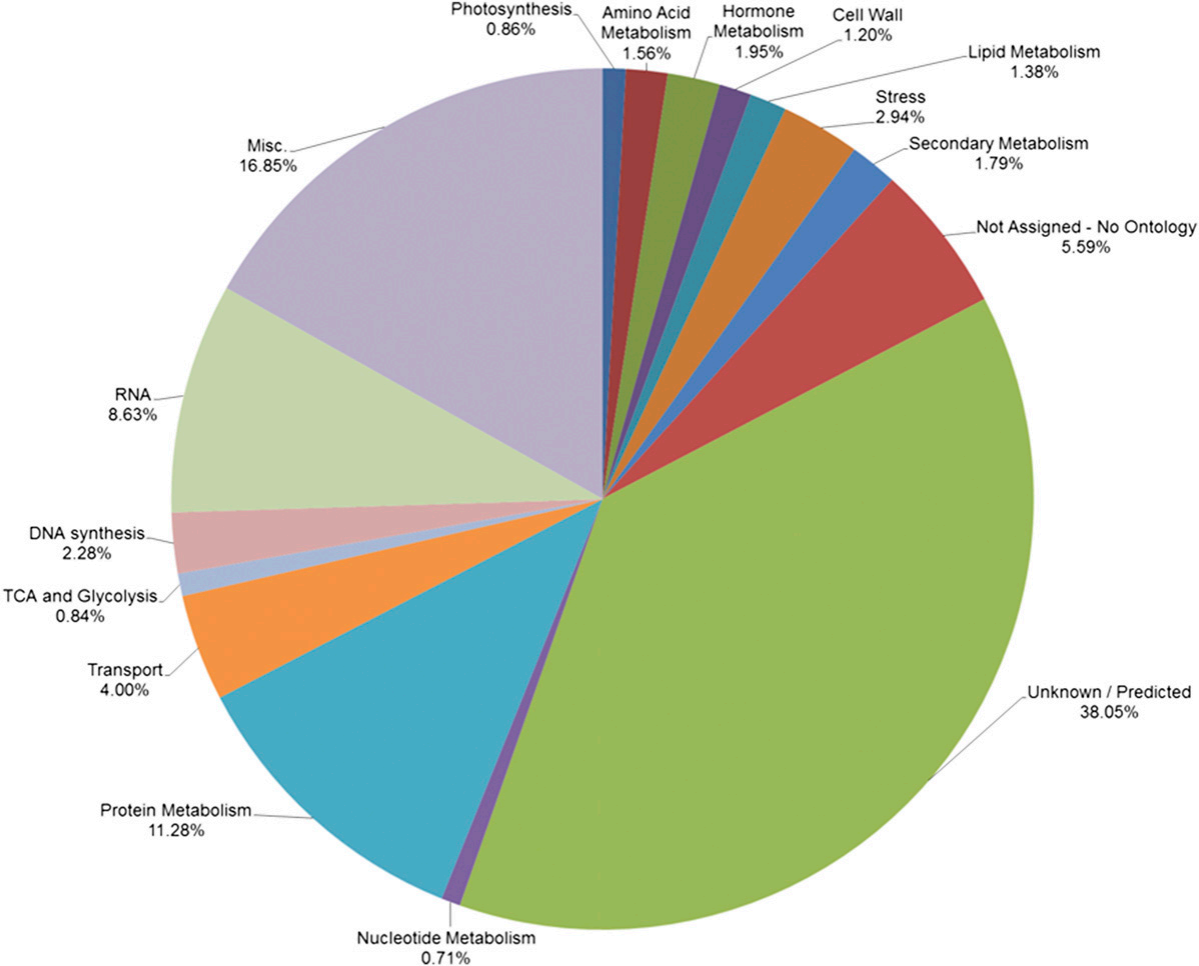
Representation of annotations of the transcripts in the consensus assembly assigned to different biological and molecular functions. (Note: Misc. signifies the transcripts that were not assigned to a category; Unknown/Predicted signifies transcripts assigned putative categories other than those listed).

**Figure 2:**
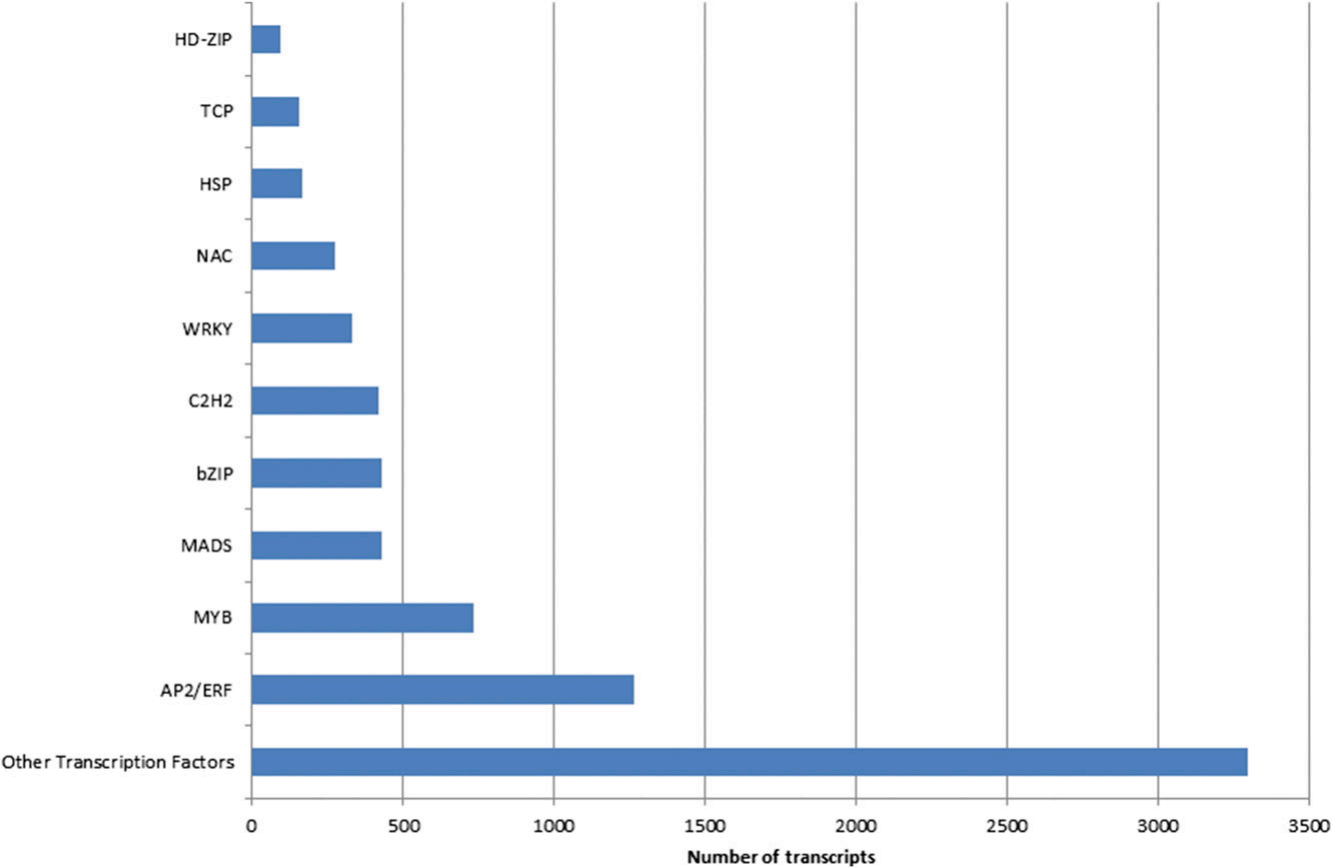
Overview of transcription factor categories in the *Arachis* consensus assembly.

A consensus assembly resulted in 165,892 contigs built from 22 transcriptomes. About 79% of the contigs from the consensus assembly were annotated, and a description of hits from each database has been deposited in Figshare. Annotated transcripts were assigned to different GO categories and this consensus assembly of *Arachis* will be a valuable resource for capturing maximum number of genes (Figure 1 and File S1). Annotation of the sequences from the consensus assembly identified numerous transcription factors. Transcription factors accounted for 4.4% of the 165,892 transcripts in the consensus assembly, and these were categorized based on their DNA-binding domain (Figure 2 and File S2). Transcription factors were assigned to different groups; b-ZIP, MADS, MYB, AP2/ERF, and C2H2, were among the top five categories (Figure 2).

### Variant analysis identified >300K SNPs

Across 22 accessions, we obtained 306,820 biallelic variants after filtering for quality parameters. SNPs were obtained by aligning raw reads of each accession to the OLin transcriptome (Chopra *et al.* 2014). On mapping the reads to this reference, the number of raw reads aligned ranged from 84 to 89%. These aligned files were then processed further using the GATK pipeline to improve the quality of SNP calls.

The lowest numbers of SNPs relative to OLin were found in the three cultivars Jupiter (3318), New Mexico Valencia C (3451), and Tamrun OL07 (3923). Landraces had more SNPs relative to OLin, ranging from 7081 to 10,832 SNPs (Table 4). SNP diversity averaged about three times higher among landraces and United States peanut minicore accessions, demonstrating significant genic diversity in the cultivated species. For *hirsuta*, and *aequatoriana*, the number of SNPs ranged from 7081 to 8212 relative to OLin (Table 4).

For the wild species, the number of SNPs relative to OLin was far higher: for A- genome diploid accessions, from 106,882 to 117,812 per accession; for B-genome diploids, from 96,241 to 109,797; for the K-genome diploids, 157,444 to 168,289; and for natural and synthetic amphidiploids, 14,340 and 87,574, respectively (Table 4). OLin reads were mapped back to the OLin reference for quality control of the variant calls.

A total of 40,382 unique SNPs was present among the cultivated accessions in 14,719 transcripts, and 291,115 unique SNPs were present among the diploid accessions in 26,320 transcripts (Table 4). The average number of SNPs among the tetraploids accessions was 0.5 per kilobase, and among the diploid accessions was 9.2 per kilobase (Table 4).

**Figure 3:**
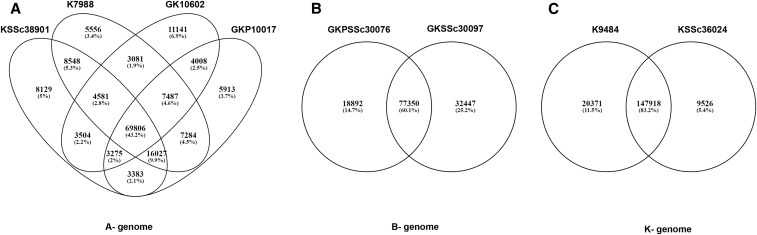
Venn diagrams representing comparison of the number of SNPs among the diploid peanut accessions. (A) A- genome. (B) B- genome. (C) K- genome. Note: Four-way and two-way comparisons were made based on the variant sites present in the accession or a group of accessions relative to the OLin transcriptome.

Many SNPs identified among accessions were unique to a genome or botanical class. A total of 191,723 SNPs was present when summed across four A- genome accessions compared to the OLin reference. We found that 43.2% of the 191,723 SNPs were shared by all four A- genome accessions, and the number of unique SNPs specific to each accession ranged from 3.4 to 6.9% (Figure 3A). Likewise, B- and K- genomes had 128,689 and 177,815 SNPs compared to OLin transcripts (Figure 3, B and C). The number of unique SNPs in *A. magna* GKSSc30097 compared to OLin was 32,430, more than the 18,092 unique SNPs between the tetraploid ancestor B- genome *A. ipaënsis* (GKPSSc30076) and OLin (Figure 3B). The number of shared unique SNPs among the K- genome accessions was 147,918 relative to OLin, while 20,371 unique SNPs belonged to *A. batizocoi* K9484, and 9526 unique SNPs to *A. cruziana* KSSc36024 (Figure 3C).

**Figure 4:**
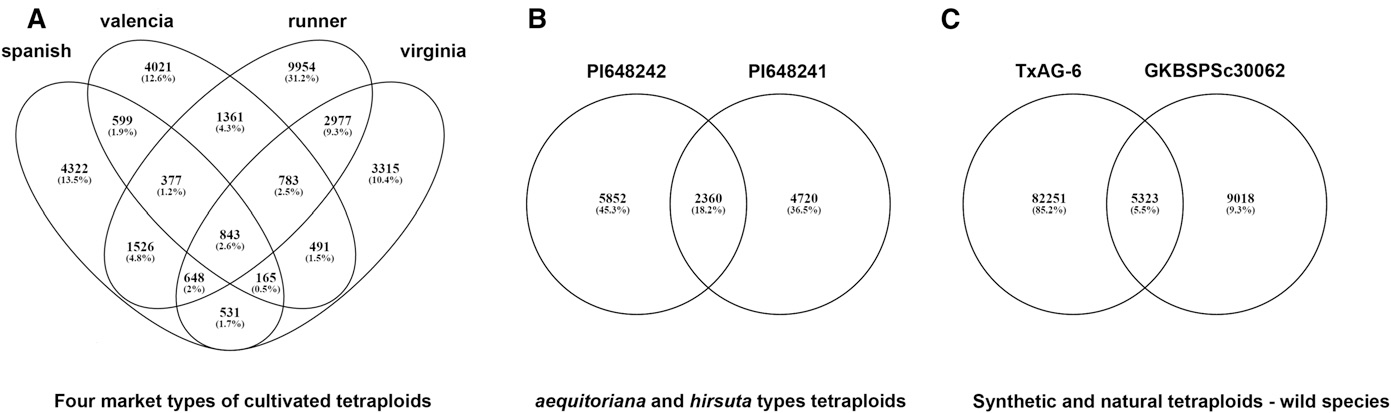
Venn diagrams representing comparison of the number of SNPs among the tetraploid peanut accessions. (A) cultivated market types. (B) other botanical types. (C) tetraploid wild species and synthetic amphidiploids. Note: Four-way and two-way comparisons were made based on the variant sites present in the accession or a group of accessions relative to the OLin transcriptome.

We found that 31,913 SNPs belonged to spanish, virginia, valencia and runners classes in comparison to the OLin transcripts (Figure 4A). For two botanical types *hirsuta* and *aequatoriana*, the number of unique SNPs was 45.3 and 36.5% of 12,932 SNPs (Figure 4B). The wild tetraploid group included an accession derived from A- and B- genome *A. monticola* (GKBSPSc30062), and the synthetic amphidiploid A- and K- genome (TxAG-6), and the differences were reflected in the number of unique SNPs (Figure 4C).

Accuracy of SNP calls was demonstrated by validation of selected SNPs by KASP marker analysis. A subset of 66 primer pairs (File S4) was designed to validate the alleles called by the bioinformatic approach, and was tested on genomic DNA of 12 sequenced accessions (File S3). A total of 782 SNP calls (12 genotypes and 66 primers) was selected, including the calls from diploid and tetraploid accessions for validation using KASP technology. Genotyping of two diploid accessions with 66 primers confirmed 88.52% of the 122 SNP calls, while 10 tetraploid accessions confirmed 80.00% of the 660 SNP calls (Table 5 and File S3).

**Table 5:** Summary of total and validated bioinformatic SNP calls among the sequenced accessions that included both diploids and tetraploids using allele-specific discrimination assays

Genotype	Diploids	Tetraploids	Total	
Homozygous	122	360	482	Total SNP calls*^b^*
Heterozygous*^a^*	—	300	300	
Total	122	660	782	
Homozygous	108	319	427	Validated SNP calls*^b^*
Heterozygous	—	209	209	
Total	108	528	636	
% matched calls	88.52	80.00	81.32	

a Heterozygous SNP calls for tetraploids are likely the result of the homeologous SNPs derived from two sub-genomes and do not indicate that genotypes are in heterozygous state.

b Total SNP calls and validated SNP calls are presented in File S3.

### Diversity was associated with population structure

**Figure 5:**
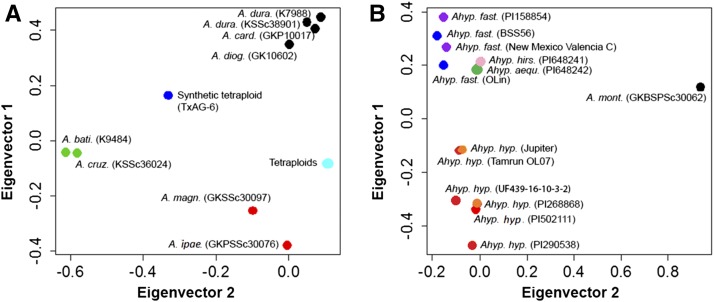
PCA using SNP data. (A) Twenty-two genotypes analyzed, which included diploids and tetraploids. Dot colors mark genome affinities of accessions as follows: black dots, A-genome accessions; red dots, B-genome accessions; green dots, K-genome accessions; blue dot, synthetic amphidiploid; cyan dot, natural tetraploids. (B) Cultivated tetraploids along with *Arachis monticola* were analyzed with diploids omitted. Dot colors mark market types of accessions as follows: violet, valencias; blue, spanish; green, aequatoriana; red, runners; orange, virginias; pink, hirsutas; black *A. monticola*.

**Figure 6:**
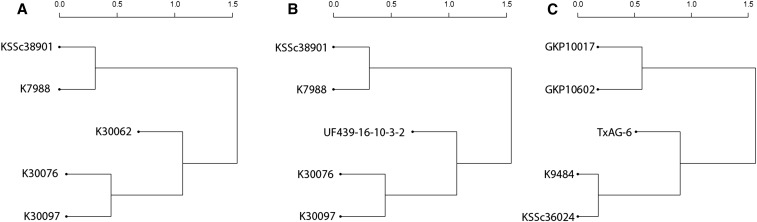
Dendrogram based on the distance matrix obtained from the polymorphism based on biallelic sites on different groups. (A) Natural tetraploids with their possible ancestors. (B) Cultivated tetraploids with their possible ancestors. (C) Synthetic tetraploid with parents from which the amphidiploid was derived.

Analysis of SNP diversity demonstrated correlation with known botanical relationships or genome affinities. We assessed genetic diversity based on the above SNPs using PCA. Eigenvector 1 (*Y* axis) identified three groups: A-genome diploids as the first group; tetraploids, TxAG-6 (synthetic tetraploid) and K- genome as the second group; and B- genome diploids as the third group (Figure 5A). Eigenvector 2 (*X* axis) separated TxAG-6, K-genome diploids, and the tetraploids from each other. Clustering of genotypes for tetraploids using PCA failed to reveal evidence of subspecies of *hypogaea*, even after the third PCA including 22 accessions. To distinguish subspecies of tetraploids, we employed the PCA approach on the same set of SNPs from the tetraploids separately, and it was possible to distinguish var *hypogaea* from var *fastigiata* (Figure 5B). From the PCA (Figure 5A), we found that the B- or K- genome is closer to the tetraploids than the A- genome, but the number of variants for diploid accessions in Table 4 suggest that the B-genome is closer to the tetraploids than are the A- or K-genome diploids. From the dissimilarity matrix (Figure 6), the B-genome species were closer to the tetraploids, and the next closer parental species belonged to the A-genome. The *hirsuta* accession did not group with var *hypogaea*, contrary to expectation; however, validation of SNP calls was carried out using seed from the Plant Introduction station using KASP markers, and the phenotype was confirmed in the field. Surprisingly, the *A. monticola* accession grouped closer to the *fastigiata* accessions than to the *hypogaea* accessions, with the exception of the *hirsuta* accession, which was closer in the first principal component.

### Phasing separated some of the merged homeologous sequences

There was evidence for significant collapse of homeologous sequences, and phasing separated a large number of merged homeologous sequences in this study. Our results among cultivars and landraces suggested that the frequency of SNPs in tetraploids was 0.5 SNP per kilobase or 0.09 per kilobase in pairwise comparisons, but we observed higher SNP rates on mapping the raw reads back to the OLin transcriptome, which could be the consequence of homeolog transcript collapse. To identify the variants in phased sequences, we aligned the sequences back to the OLin reference transcriptome. There were a total of 25,488 contigs (53.4%) that contained >1 SNP, which were therefore good candidates for polymorphism phasing (Table 6). Assuming that >1 SNP per transcript could be a result of homeolog collapse, we selected transcripts with >1 SNP to separate transcripts using the HapCUT program. We phased SNPs in 22,683 contigs containing >1 SNP into 30,672 sub-transcripts, resulting in an average of 1.35 separated transcripts per contig. During the readphaser process, only 10,815 of 22,683 phased contigs were carried forward for assembly, as the remaining contigs had either evidence of a third haplotype or low read support. Phased assemblies consisted of a total of 81,244 contigs, which were further used to evaluate the separation of homeologs.

**Table 6:** Summary of the assembly and SNP calls using *de novo* and phased approach on OLin transcriptome

Name	No. of Contigs	N50	Contigs with SNPs	Contigs with >1 SNPs	Contigs with >2 SNPs	Heterozygous Calls*^a^*
OLin_*de novo*	67,098	1641	30,918	25,888	22,071	241,497
OLin_Phased	87,214	1292	29,677	20,440	14,593	109,937

a Heterozygous calls could be the result of homeolog collapse in the reference or alignment errors.

The phasing strategy generated higher numbers of contigs than the *de novo* assembly approach, but had lesser representation of the transcriptome, as only 76–78% of the reads mapped back. Variant calls from phased assemblies indicated a sharp decrease in the number of heterozygous SNPs, and the number of contigs with multiple SNPs per transcript was lower compared to the *de novo* assembly (Table 6). The number of contigs with >2 SNPs was reduced by ∼33%, while the number of contigs with >1 SNP was reduced by 20% in the phased assembly approach compared to the *de novo* approach.

## Discussion

### Significant genic diversity exists in peanut

SNP diversity analysis demonstrated the presence of significant genic diversity among cultivated accessions and wild species. The number of SNPs identified across all 22 genotypes suggested less genetic variation among *A. hypogaea* accessions compared to the wild diploids, which had substantially higher genetic variation. High phenotypic diversity observed among diploid accessions is consistent with genetic variation identified in comparison to the OLin reference. For the United States cultivars, the number of homozygous SNPs was in the range of 3000–3500, which agreed with the data previously reported by Chopra *et al.* (2015). Among the landraces of tetraploid peanut, homozygous SNPs ranged from 7000 to 10,000 SNPs, double to triple the number in United States cultivars. Such a high number of SNPs among the tetraploid accessions suggested high genic diversity exists compared to previous reports of low, or no, molecular variability (Kochert *et al.* 1996; Burow *et al.* 2009) using RFLP markers. Also, this genic variability among the landraces could reflect the level of phenotypic diversity observed among core and minicore collections of peanut. Indeed, four of the accessions screened in this study were chosen from the United States peanut minicore collection. Previous minicore screens have also shown that there is huge variability for traits such as water use efficiency (Singh *et al.* 2014), oil content (Wang *et al.* 2011a,b), bacterial wilt disease (Jiang *et al.* 2014), resistance to abiotic and biotic stresses (Upadhyaya *et al.* 2013), and agronomic traits (Pandey *et al.* 2014). Sufficient variation has been identified among tetraploid accessions through transcriptome sequencing for performing QTL analysis with high density marker linkage maps.

To call SNPs on 22 accessions, we aligned raw reads to the reference (OLin). The raw reads aligning to the reference ranged from 84 to 89%, suggesting that the assembly used in this study had a good representation of the peanut transcriptomes. A total of 520 SNPs was identified by comparing the OLin transcriptome to the reference assembly; this may suggest the presence of gene copy number variation, call errors, or artifacts in the software used. The presence of SNPs when mapping reads back to the same reference has been cited previously (Nielsen *et al.* 2011).

The SNP rate in tetraploid peanuts identified was 0.5 per kilobase, which is lower than other related legume species such as soybean (2.7 SNPs per kilobase) (Choi *et al.* 2007), field pea (2.7 SNPs per kilobase) (Leonforte *et al.* 2013), and *Medicago truncatula* (1.96 SNPs per kilobase) (Choi *et al.* 2004). However, in diploid species of peanut, including different genome affinities, SNP rates reported here were higher than in other legumes.

### Phylogenetic analysis demonstrated that SNP diversity is associated with population structure

Previous genetic diversity analyses have indicated clear differentiation of diploid species and tetraploid subspecies (Burow *et al.* 2009). Based on thousands of SNPs identified in this study, we also differentiated tetraploids from the diploids (Figure 3A), and individuals of the two subspecies of *A. hypogaea* (Figure 3B). The number of SNPs differentiating accessions of *A. hypogaea* and *A. monticola* was also higher compared to differences among cultivars, providing evidence of additional untapped genetic diversity.

In general, the *hypogaea* accessions grouped together; likewise the *fastigiata* accessions. Two unusual results were in the first PCA results, in which the *hirsuta* accession and *A. monticola* were closer to each other and to the *fastigiata* accessions. This is surprising, because the spreading type of these accessions matches the *hypogaea* accessions. However, there are other phenotypic data to consider—for example, the pronounced reticulation of pods of *hirsuta*, *peruviana*, and *aequatoriana* accessions resembles each other more than the typical *hypogaea* or *fastigiata* accessions. As with the case of the greater similarity of *A. monticola* to the *fastigiata* type, inclusion of data from other accessions in a separate study would be warranted before making broader conclusions, as the present data are based on only a single accession.

### Validation of SNPs demonstrated high potential utility for marker-assisted breeding using KASP markers

Another goal of this research was to identify SNP markers that could be useful in breeding programs. Of the 782 selected SNP calls tested, 81% were confirmed on the LightCycler 480, which is significantly better than results of previous studies in peanut (Khera *et al.* 2013; Chopra *et al.* 2015). The two diploid accessions used for validation were selected to demonstrate the approach used for identification of SNPs in peanut, and for development of a genetic map (R. Chopra *et al.*, unpublished results). Tetraploid accessions were used to confirm the SNP calls that could be eventually used in population studies. The overall validation success rate of 81.32% of 782 selected SNP sites was much higher than in a previous study by Chopra *et al.* (2015). Validation using KASP chemistry suggested that the variant calling approach is effective in diploid and tetraploid peanut. This provides a basis for selecting SNP markers between the parents for future breeding activities.

Annotations of the transcripts in the consensus assembly and the OLin assembly by Chopra *et al.* (2014) provided evidence of SNPs in important trait-related transcripts. For example, SNPs were observed in oil biosynthesis genes, and in the genes of QTL regions affecting nematode resistance. Diagnostic KASP markers were designed and are being evaluated in the breeding program (R. Chopra *et al.*, unpublished results). Collectively, these datasets have the potential for enabling SNP identification throughout the genome for any polymorphic gene of interest, thus making the tagging of specific genes with molecular markers a possibility.

### Unique genes per peanut genome are in the range of 25–32K

Results of *de novo* assembly suggest that there are about 25,000–31,500 unique transcripts in diploid and tetraploid *Arachis* roots, leaves, and developing pod tissues. This number could be a minimum value, as not all tissues were sampled. The number of transcripts in tetraploids could be twice that of diploids because of homeologous copies. Given the allo-tetraploid nature of the peanut, genes from both genomes are similar, and tools used in the study in general could not distinguish homeologous genes from each other. Consensus assemblies had more contigs than any other assembly by itself, which suggested that each genotype has a certain percentage of transcripts that could not be merged at a minimum sequence similarity threshold of 95%. This increase in the number of transcripts could be due to the larger differences among species/accessions used, or differences in expression of transcripts in samples. For tetraploids, the increase in number of transcripts in the consensus assembly can also be a result of the presence of homeologs. Consensus and individual assemblies from this study could benefit annotation of the genome for rare transcripts and gene expression studies.

### Phasing of homeologous copies requires longer reads

In the case of diploids, the success rate of SNP validation was greater than for tetraploid calls. Complexity of the tetraploid genome resulting from homeologous copies may have affected the outcomes of validation. Based on previous studies in complex polyploids, such as wheat (Schreiber *et al.* 2012) and peanut (Chopra *et al.* 2014), it was observed that a significant number of homeologs would be merged even after optimizing the *de novo* assembly parameters. Tetraploid SNP calls consisted of many apparently heterozygous state genotypes, and to reduce these heterozygous calls, we evaluated the phasing approach. We utilized the postassembly approach reported by Krasileva *et al.* (2013) to separate merged homeologous transcripts with assemblers. This approach gave a more complete overview of the tetraploid transcriptome with a smaller number of chimeric transcripts. Ideally, mapping the reads back to the reference derived from same accession should give fewer variant calls, but, due to collapse of some homeologs, the heterozygous variant calls were more in the *de novo* assembly. One way to estimate the reduction of homeolog collapse would be to observe the number of heterozygous variants upon mapping the reads back. Reduction in heterozygous SNP calls compared to the reference would be the first step for improving the assembly qualities.

After evaluating the results of resolving homeologs, we suggest that, to get a complete separation of transcripts with no collapse would require: (a) longer sequence reads, because shorter reads can affect the extension of contigs with the poor quality on the ends of reads; (b) higher read coverage to support the haplotypes, because lower read depth haplotypes could be dropped by the phasing based approach; and (c) using the genome sequence of diploid progenitor species to avoid collapse, and to obtain a better representation of transcripts.

### Conclusions

We report the first survey of significant SNP level and transcript level diversity in a wide array of peanut germplasm. Highlighted untapped genetic variability among accessions may explain some of the phenotypic differences observed in germplasm surveys. Transcriptome analysis among cultivated accessions, and comparison to wild species will open many avenues for future research. Among these are relationships of cultivated tetraploid species to their diploid ancestors, evolutionary development of the cultigen, distinguishing of homologous *vs.* homeologous SNPs for genetic mapping and QTL analysis, study of genome rearrangements after polyploidization, and expression bias among genes and genomes. Outcomes of this research will help increase of genomic resources in peanut, and benefit QTL mapping relevant for agronomic traits.

## Supplementary Material

Supplemental Material
