# Supplementary material for: Transcriptome Sequencing of Diverse Peanut (Arachis) Wild Species and the Cultivated Species Reveals a Wealth of Untapped Genetic Variability
Source: G3 (Bethesda). 2016 Oct 10;6(12):3825–36. doi: 10.1534/g3.115.026898 (PMC5144954; doi:10.1534/g3.115.026898)
Supplement: Supplemental Material [file supp_6_12_3825__index.html]

Transcriptome Sequencing of Diverse Peanut (Arachis) Wild Species and the Cultivated Species Reveals a Wealth of Untapped Genetic Variability — Supplemental Material 

# Transcriptome Sequencing of Diverse Peanut (*Arachis*) Wild Species and the Cultivated Species Reveals a Wealth of Untapped Genetic Variability

## Supplemental Material for Chopra *et al.*, 2016

**Files in this Data Supplement:**

- File S1 - Brief annotations of the transcripts in the consensus assembly assigned to different biological and molecular functions by Mercator. (.xlsx, 4 MB)
- File S2 - Transcription factor categories in the *Arachis* consensus assembly. (.xlsx, 733 KB)
- File S3 - Primer sequences used in validation of bioinformatic calls in twelve of the sequenced accessions. (.xlsx, 13 KB)
- File S4 - Selected SNP calls among twelve accessions and genotype calls confirmed using allele-specific discrimination assay (KASP). (.xlsx, 31 KB)
